# The Potential of Raman Spectroscopy in the Diagnosis of Dysplastic and Malignant Oral Lesions

**DOI:** 10.3390/cancers13040619

**Published:** 2021-02-04

**Authors:** Ola Ibrahim, Mary Toner, Stephen Flint, Hugh J. Byrne, Fiona M. Lyng

**Affiliations:** 1 School of Dental Science, Trinity College Dublin, Lincoln Place, D02 Dublin 2, Ireland; 2 Central Pathology Laboratory, St. James Hospital, James Street, D08 Dublin 8, Ireland; mtoner@stjames.ie; 3 Oral Medicine Unit, Dublin Dental University Hospital, Trinity College Dublin, Lincoln Place, D02 Dublin 2, Ireland; stephen.flint@dental.tcd.ie; 4 FOCAS Research Institute, City Campus, Technological University Dublin, Kevin Street, D08 Dublin 8, Ireland; hugh.byrne@tudublin.ie; 5 Radiation and Environmental Science Centre FOCAS Research Institute, City Campus, Technological University Dublin, Kevin Street, D08 Dublin 8, Ireland; fiona.lyng@tudublin.ie; 6 School of Physics & Clinical & Optometric Sciences, City Campus, Technological University Dublin, Kevin Street, D08 Dublin 8, Ireland

**Keywords:** oral cancer, oral pre-cancer, oral dysplasia, premalignant lesions, potentially malignant lesions, Raman spectroscopy

## Abstract

**Simple Summary:**

Raman spectroscopy, a light scattering technique that provides the biochemical fingerprint of a sample, was used on samples taken from patients with cancer and precancerous lesions. This information was then used to build a classifier to identify cancer and the precancerous phases. The ability to distinguish cancerous tissue from normal and precancerous tissue is diagnostically crucial as it can alter the patients’ prognosis and management. Moreover, as cellular changes are often present at the tumour margin, the ability to distinguish these changes from cancer can help in preserving more of the tissue and maintaining aesthetics and functionality for the patient.

**Abstract:**

Early diagnosis, treatment and/or surveillance of oral premalignant lesions are important in preventing progression to oral squamous cell carcinoma (OSCC). The current gold standard is through histopathological diagnosis, which is limited by inter- and intra-observer errors and sampling errors. The objective of this work was to use Raman spectroscopy to discriminate between benign, mild, moderate and severe dysplasia and OSCC in formalin fixed paraffin preserved (FFPP) tissues. The study included 72 different pathologies from which 17 were benign lesions, 20 mildly dysplastic, 20 moderately dysplastic, 10 severely dysplastic and 5 invasive OSCC. The glass substrate and paraffin wax background were digitally removed and PLSDA with LOPO cross-validation was used to differentiate the pathologies. OSCC could be differentiated from the other pathologies with an accuracy of 70%, while the accuracy of the classifier for benign, moderate and severe dysplasia was ~60%. The accuracy of the classifier was lowest for mild dysplasia (~46%). The main discriminating features were increased nucleic acid contributions and decreased protein and lipid contributions in the epithelium and decreased collagen contributions in the connective tissue. Smoking and the presence of inflammation were found to significantly influence the Raman classification with respective accuracies of 76% and 94%.

## 1. Introduction

Oral cancer (OC) is the 16th most common cancer worldwide, 354,864 new cases and 177,384 deaths having been reported in 2018 [1]. Over 90% of oral cancers are squamous cell carcinomas affecting the tongue, floor of the mouth, lips, gingivae, buccal mucosa and palate. The major risk factors for developing oral cancer are smoking and alcohol consumption, which can work synergistically [2,3,4]. Premalignant lesions such as leukoplakia (white patch) and erythroplakia (red patch) carry an increased risk of malignant transformation [5]. Different degrees of dysplasia can be found in the premalignant lesions which are classified into hyperplasia, mild, moderate and severe dysplasia and carcinoma in situ, depending on the degree of architectural disturbance and cytologic atypia [5]. Generally, 5–25% of oral leukoplakia are dysplastic, while almost all erythroplakia show some degree of dysplasia [5]. Despite advances in therapeutic management, there has been no significant improvement in the 5 year survival rate of OC, which remains at around 50% [6]. This, in part, is due to the fact that over 40% of patients present at an advanced stage, at which nodal involvement and distant metastasis have occurred [7]. This highlights the importance of early diagnosis. Currently the gold standard for diagnosing OC and dysplasia is through a conventional clinical oral examination, followed by a biopsy of any suspicious lesions and their histopathological examination [8]. The issue with this method is that it is subjective and prone to inter- and intra- observer errors [9]. Additionally, a biopsy may not be representative of the whole lesion, as studies looking at the histology of tumours post operatively and comparing them to the preoperative biopsies have found that, in a significant number of cases, a neoplasia or carcinoma in-situ was misdiagnosed [10].

Raman Spectroscopy is a technique that was developed based on the Raman effect. When electro-magnetic (EM) radiation interacts with a sample, it may be absorbed, or scattered. While most scattering is elastic, named Rayleigh scatter, the Raman effect describes the inelastic scattering that occurs in a small number of photons (about 1 in a million), which lose or gain energy by interaction with the material vibrations. The Raman scattered light can be collected by a spectrometer and displayed as a Raman spectrum, in which the peaks (bands) correspond to Raman frequency shifts (measured in wavenumbers cm^−1^) caused by the characteristic vibrations in the molecules of a sample. There has been a lot of interest in the use of Raman spectroscopy in medical diagnostics since its introduction to the field almost 30 years ago [11]. Its advantages, such as minimal sample preparation, speed, non-invasiveness, label free nature, and the fact that it gives both qualitative and quantitative information on the molecular content of a sample make it particularly suited to such applications. Over the past 20 years, there have been numerous studies in the area of Raman spectroscopy for diagnosis of a wide range of cancers, including breast, lung, prostate, cervical, oesophageal and colon (reviewed in [12,13,14,15,16].) These studies demonstrate that Raman spectroscopy can be used to distinguish the different stages in the progression of a cell from normal to cancerous. Monitoring cancer progression after the withdrawal of carcinogens is another avenue that has been explored using Raman spectroscopy [17]. In addition, Raman spectroscopy has recently been shown to have potential for screening for metastases [18,19] and for companion diagnostics [20]. There have been several studies on Raman spectroscopy for oral cancer, and the state of the art and challenges have been recently reviewed [21]. Notably, however, there has been very little work on oral dysplasia or oral pre-cancer. Using OSCC and dysplastic cell lines and comparing them to normal cells, a study has found that Raman spectroscopy could discriminate between malignant, dysplastic and normal cells in the fingerprint region based on varying nucleic acid, protein and lipid profiles [22]. Similar results were obtained from the high wavenumber region of the spectrum [23]. Studies on fresh and frozen tongue tissue sections could classify OSCC from normal tissue using Raman spectroscopy with a high degree of accuracy [24,25,26,27]. Analysis of the water content in the high wavenumber region of spectra obtained in OSCC bone resection margins, classified OSCC from healthy tissue with 95% accuracy [28]. Nevertheless, a study looking at surgical margins in sections of OSCC found that the accuracy of the Raman classification for dysplastic tissue was only 48% [29].

In the present study, we aimed to assess whether Raman spectroscopy can discriminate between benign lesions, different degrees of dysplasia and OSCC from the biopsied tissues of a cohort of patients and to evaluate the influence of patient factors and clinical features on the Raman spectra of the tissues.

## 2. Results

### 2.1. Epithelial Tissue

**Figure 1:**
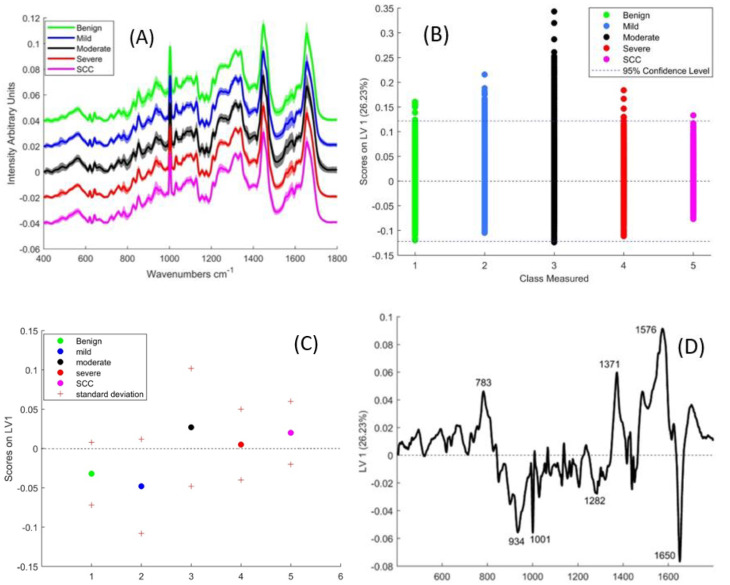
(**A**) Mean Raman spectra of benign, mild, moderate and severely dysplastic epithelial tissue. The spectra have been offset for clarity and shading denotes standard deviation (**B**) A plot of the PLSDA scores according to LV-1 (**C**) Mean and standard deviation of PLSDA scores of LV-1 (**D**) LV-1 of the PLSDA model for Epithelial tissue, including all the classes.

Figure 1A shows the mean Raman spectra of epithelial tissue in each cohort. Table 1 lists the concurrent peak assignments [30].

The results of the partial least squares discriminant analysis (PLSDA) classification do not show a very good discrimination across the groups (Table 2). The estimated ROC curves are based on predicted class for each spectrum. Sensitivity is calculated from the fraction of in-class spectra while the specificity is calculated from the fraction of not-in-class spectra for a given threshold. The cross validated ROC curves follow the same method, except the class predicted when the spectra are left out during cross validation is used. From the ROC curves (Figure S1), it appears that the classifier has the highest accuracy for SCC (AUC = 0.71) and lowest for mildly dysplastic epithelium (AUC = 0.46).

To better elucidate the variability between the different classes, their scores on the first latent variable (LV-1) were plotted. This shows a large intra-class spread, the greatest spread being observed in the moderate group and the smallest in the SCC group (Figure 1B). Plotting the means and standard deviations of the scores on LV-1 (Figure 1C) does not show an obvious progression, but it can be assumed from their means that the benign and mild are mostly negative for LV-1, while moderate, severe and SCC are mostly positive. LV-1 (Figure 1D), which is reponsible for 26.23% of the variance, has positive peaks at 783, 1371 and 1576 cm^−1^, which relate to nucleic acids (Table 1). Negative peaks are observed at 934, and 1282 cm^−1^ (relating to protein/collagen) and the amide 1 band at 1650 cm^−1^.

### 2.2. Connective Tissue

**Figure 2:**
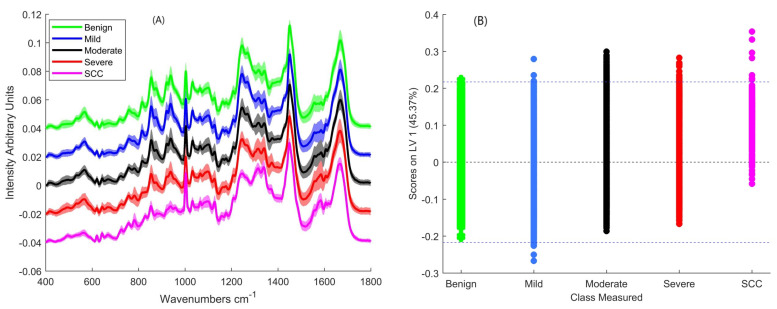

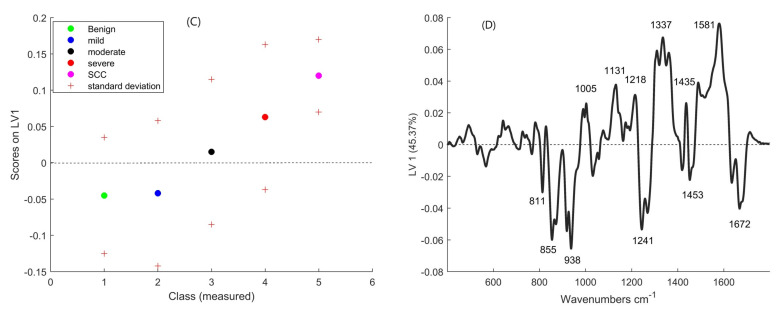
(**A**) Mean Raman spectra of benign, mild, moderate severely dysplastic and SCC connective tissue. The spectra have been offset for clarity and shading denotes standard deviation (**B**) A plot of the PLSDA scores of LV-1 (**C**) Mean and standard deviation of PLSDA scores of LV-1 (**D**) Loading of LV-1 of the PLSDA model which included all the classes.

From their mean spectra (Figure 2A), the most notable difference between benign, mild, moderate, severe and SCC connective tissue appears to be in the regions 800–1000 cm^−1^ which correspond to different collagen assignments and 1200–1400 cm^−1^ which correspond to vibrations in lipids, nucleic acid bases, and collagen (Table 1). The results of the PLSDA classification (Table 2) show high sensitivities for benign and SCC compared to the dysplasia classes. However, the specificity for benign was low, indicating a high false positive rate. The classifier has the best accuracy among the classes for SCC according to the ROC curve (Figure S2).

Plotting the scores of LV-1 shows the greatest intra-class spread in the mild group and the smallest in the SCC group (Figure 2B). Plotting the means and standard deviations of the scores of LV-1 (Figure 2C) shows a progression from benign to SCC on LV-1. The means of the benign and mild are negative in LV-1 while those of moderate, severe and SCC are positive.

Positive peaks of LV-1 can be observed at 1005, 1131, 1218, 1337, 1435 and 1581 cm^−1^ LV-1 (Figure 2D). The peaks at 1005 and 1581 cm^−1^ relate to phenylalanine, while those at 1131, 1218 and 1435 cm^−1^ relate to lipids and that at 1337 cm^−1^ relates to nucleic acids. On the other hand, negative peaks can be observed at 811, 855, 938, 1241, 1453 and 1672 cm^−1^. The peaks at 855, 938 and 1241 cm^−1^ relate to collagen while 1453 and 1672 cm^−1^ relate to lipid contributions.

### 2.3. Influence of Patient Factors and Clinical Features on Raman Classification

Other factors which could have an influence on the Raman classification were assessed. Metadata was used to divide all the patients, regardless of histopathological diagnosis, into groups according to gender, smoking habits, alcohol consumption, site of lesion and presence of inflammation. A total of two factors were found to influence the Raman classification, namely smoking and the presence of inflammation.

#### 2.3.1. Smoking

The patients were divided into three groups according to smoking status; non-smoker, ex-smoker (previous smokers) and smoker (Table 3).

The PLSDA results showed high classification sensitivity for non-smokers and ex-smokers but lower specificities. On the other hand, the classification sensitivity was lower for smokers but the specificity was higher (Table 3). The ROC curve (Figure S3) shows a significant accuracy (AUC = 0.76) of the classifier for smokers.

**Figure 3:**
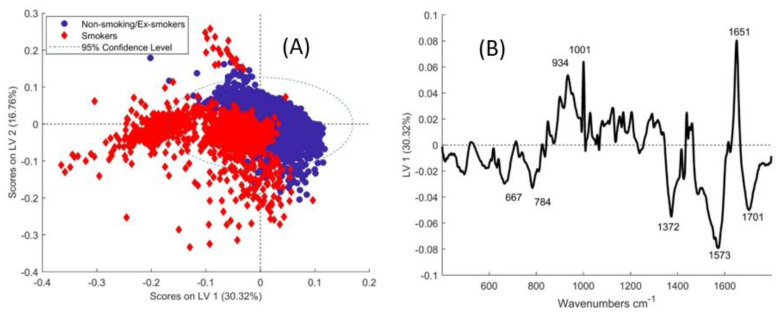
(**A**) Scores of smokers and non-smoker/ex-smokers on the latent variables from the PLSDA model. (**B**) Loading of LV-1 from PLSDA of smokers vs. non-smoker and ex-smokers in epithelial tissue.

To further understand the source of the variance, non-smokers and ex-smokers were combined and the scores of LV-1 and LV-2 were plotted against those for smokers (Figure 3A). While there is some overlap, smokers are mainly negative in LV-1, while non-smoker/ex-smokers are mainly positive. According to LV-1, negative bands at 667, 784, 1372, and 1573 cm^−1^ suggest higher levels of nucleic acids in the epithelium of smokers. Non-smoker/ex-smokers had a more prominent amide I band at 1651 cm^−1^ and protein band at 934 cm^−1^ (Figure 3B).

#### 2.3.2. Presence of Inflammation

All the pathologies were evaluated for the presence of inflammation, indicated by the presence of inflammatory infiltrate with chiefly lymphocytes and mast cells. The H & E stained slides were evaluated under a bright-field microscope. Table 4 shows the number of inflamed samples per class. PLSDA was used to classify inflamed vs. non-inflamed for all the pathologies combined. The results show that inflamed tissue can be classified from non-inflamed tissue with sensitivity and specificity values of 68% and 70%, respectively, in epithelium and 77% and 86%, respectively, in connective tissue. The AUCs were significant, 0.72 for epithelium and 0.84 for connective tissue (Figure S4).

**Figure 4:**
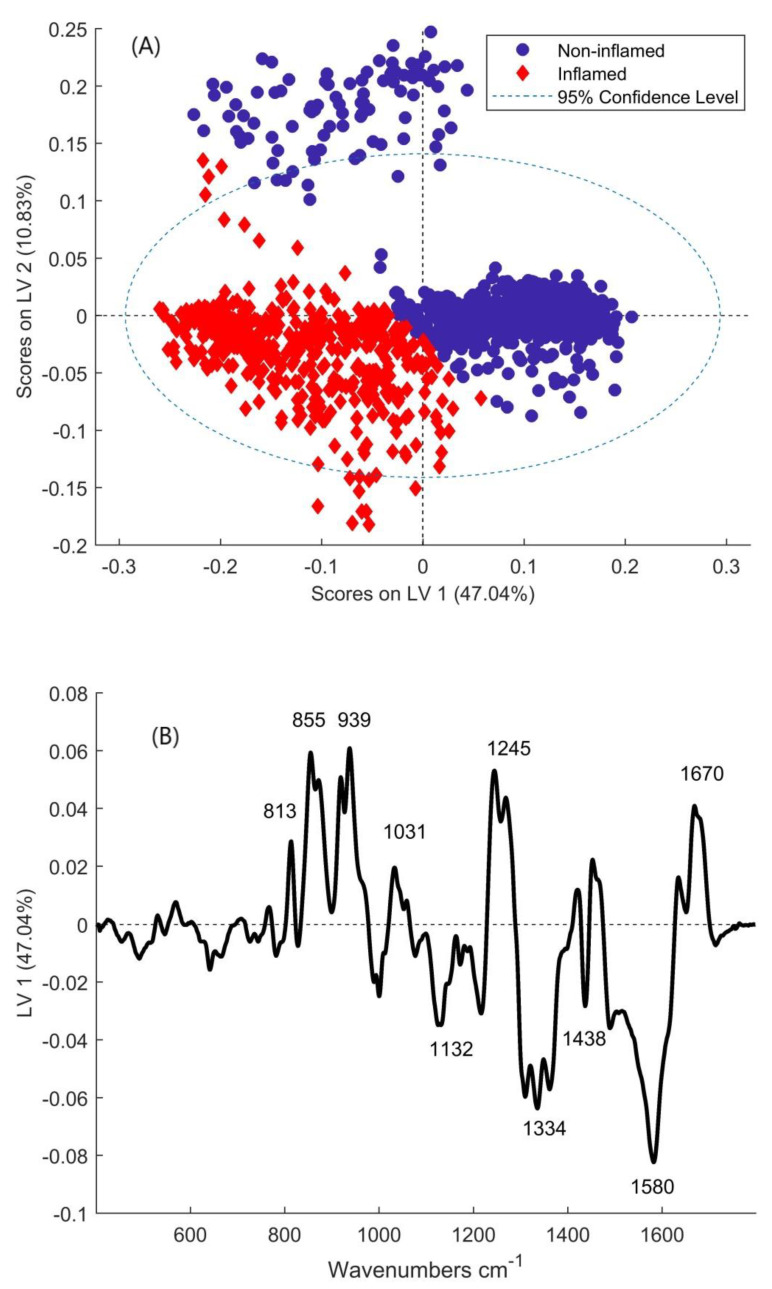
(**A**) Scores of inflamed and non-inflamed moderately dysplastic connective tissue on the latent variables from the PLSDA model. (**B**) Loading of LV-1 from the PLSDA model of inflamed vs. non-inflamed connective tissue.

To ensure that the results obtained are due to the presence of inflammation rather than the pathology (as most of the severe and SCC samples were inflamed, which could skew the results), inflamed vs. non-inflamed was assessed in the moderate category. The results show a very high accuracy in connective tissue (AUC = 0.94) and, to a lesser extent, in epithelium (AUC = 0.69) (Figure S5). Plotting the scores of the latent variables shows a good separation based on LV-1, the majority of inflamed spectra have negative scores while the majority of non-inflamed spectra have positive scores on LV-1 (Figure 4A) The group of non-inflamed spectra that are outside the 95% confidence interval are likely from one patient who was misclassified due to increased variability from the rest of the non-inflamed group. The loading of LV-1 (Figure 4B) shows positive peaks at 813, 855, 939, 1031, and 1245 cm^−1^ which relate to collagen (Table 1) while the negative peaks relate to nucleic acids (1334, 1580 cm^−1^) and fatty acids (1132, 1438 cm^−1^).

## 3. Discussion

Raman spectroscopy can uncover a wealth of biochemical information including the lipid, protein and nucleic acid content of the tissue, which in turn can reflect the presence and degree of tissue pathology.

The choice to study each part of the tissue (epithelium and connective tissue) independently was made in order to better understand/identify the changes taking place in each. While it was expected to find discrimination between severe, mild and moderate in the epithelium, as the epithelial cells are undergoing morphological and biochemical changes, significant differences in connective tissue between the pathologies were not expected.

Results from the PLSDA show increasing nucleic acid contributions and lower protein and lipid contributions as dysplasia progresses in the epithelium. According to the ROC curves, the accuracy of the classifier was highest for the SCC class (AUC = 0.71), intermediate (AUC~0.6) for the benign, moderate and severe classes, and lowest (AUC = 0.46) for the mild, resulting in misclassification with benign and moderate. The moderate group had the lowest sensitivity in the PLSDA classification and the greatest spread in LV-1, suggesting a higher variability in this group compared to the others. It is important to note that these classifications are based on histological grading by one pathologist, whereas Raman spectroscopy measures the biochemical composition of the sample. Hence incipient biochemical changes before the onset of tissue morphological changes might be influencing the classification.

In connective tissue, nucleic acid peaks were more prominent with progressive dysplasia and collagen peaks were less prominent. Connective tissue associated with SCC could be classified from that associated with dysplasia and with benign lesions with a high sensitivity and specificity. This is to be expected as, due to epithelial mesenchymal transition [31]; the boundary between epithelium and connective tissue in SCC is often lost as a result of islands of epithelium invading the connective tissue [32].

From the results, it is apparent that some factors other than the degree of dysplasia can influence the Raman classification. While it has been reported that age related physiological changes can be discriminated with Raman spectroscopy [33,34], most of the patients in this cohort were between 50–60 years old, and hence there was not enough variation to study age related factors. No discrimination based on gender was apparent; the female vs. male sensitivity and specificity values in epithelium were 22% and 77%, respectively. In connective tissue, the sensitivity was 62% and specificity was 44% (Figure S6). Other patient factors and clinical features which have not been considered, due to lack of metadata, could potentially have an influence on the Raman classification. These include HPV and candida status of the patients, the size of the lesions, and the degree of differentiation in the SCC lesions.

Smoking status was seen to impact on the classification of epithelial tissue (AUC = 0.76). This is consistent with previous work by Singh et al., who have shown that the oral buccal mucosa of smokers is more likely to misclassify with that of premalignant lesions than that of non-smokers [33,35]. This is likely due to the fact that smoking is an aetiological factor in developing oral dysplasia, and hence biochemical changes occurring in the mucosa of smokers are similar to those occurring in dysplastic lesions.

The presence of inflammation in connective tissue, however, was found to have a significant influence on the Raman classification (AUC = 0.94). Reduced collagen features and increased nucleic acid features in the Raman spectra of inflamed connective tissue were the main findings and this has been previously shown for cervical tissue [36]. The nucleic acid features may be due to increased cellularity caused by the inflammatory cells infiltrating the tissue. The reduction of collagen features is likely due to the breakdown of collagen by matrix metalloproteinases (especially MMP-8) which are upregulated in inflammation [37]. In this study, most of the severely dysplastic and SCC tissue was found to be inflamed, which is consistent with a previous study that has shown increasing inflammatory cell infiltration with increasing severity of oral dysplasia and SCC [38]. The presence of inflammation in the tumour microenvironment has been well documented and is due to multiple factors [39,40]. The environmental factors that prompt carcinogenesis, such as alcohol and smoking, have been shown to trigger an inflammatory response [41]. Furthermore, the tumour cells release inflammatory mediators which generate an inflammatory microenvironment that promotes cancer growth, invasion and metastasis [42]. A study looking at OSCC surgical margins found that inflamed connective tissue was more likely to misclassify with SCC than non-inflamed connective tissue [29].

## 4. Materials and Methods

### 4.1. Sample Preparation

**Figure 5:**
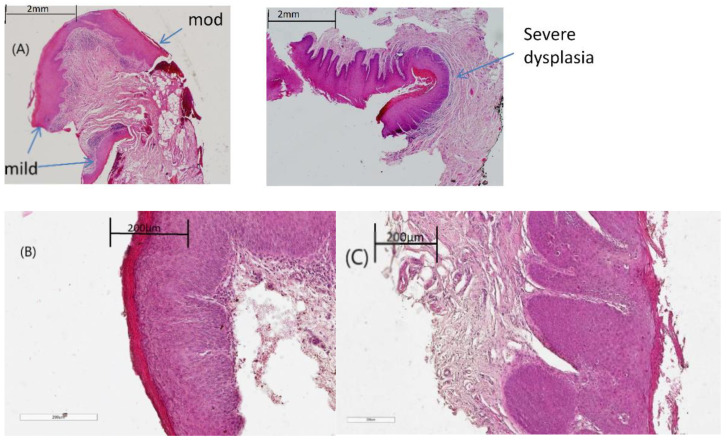
Representative H & E images showing (**A**) the regions of dysplasia marked by the pathologist. (**B**) A magnified region of moderate dysplasia (**C**) A magnified region of severe dysplasia. Scale bar: 200 µm.

Archival oral formalin fixed paraffin preserved (FFPP) tissues for each patient cohort were obtained following ethical approval from St James’ Hospital Ethics Committee and informed written consent from patients. The haematoxylin and eosin (H & E) stained sections from the different pathologies were examined by a pathologist and the areas of interest were annotated. In total, 57 patients were included, from which 72 pathologies were identified, including 17 benign lesions, 20 mildly dysplastic, 20 moderately dysplastic, 10 severely dysplastic and 5 invasive SCC. The FFPP tissue blocks and corresponding images were then taken to the laboratory, where 10 µm sections were cut from the FFPP tissues and mounted on glass slides. One of the sections from each sample was dewaxed, stained with H & E (Figure 5), and a parallel unstained section was used for Raman spectroscopic measurement.

### 4.2. Instrumentation

**Figure 6:**
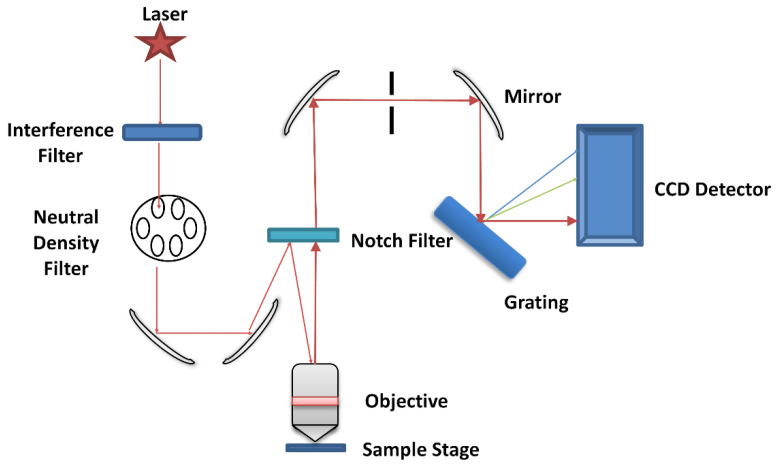
A schematic of a Raman microspectrometer based on the Horiba Jobin Yvon LabRAM HR 800.

A confocal, Horiba Jobin Yvon LabRam HR 800 Raman (upright) spectroscopic microscope (Figure 6) was used to record the spectra of the FFPP oral tissue. The microscope has an automated xyz stage and is coupled to a Peltier cooled CCD detector. A 50 mW diode laser of 532 nm wavelength was used and the grating was set at 600 grooves/mm, while the confocal hole was set at the recommended 100 µm. For mapping acquisition, the regions to map were selected using a 100× objective (MPLAN N Olympus, Japan, NA = 0.9, spot size ~1 m), which also collected the backscattered light. The spectra were acquired over two accumulations, totalling 20 s per spectrum. The step size was set at 10 µm and the spectral range was 400–1800 cm^−1^. For every pathology section, 200 spectral points were taken from epithelium and the same from connective tissue.

### 4.3. Data Analysis

**Figure 7:**
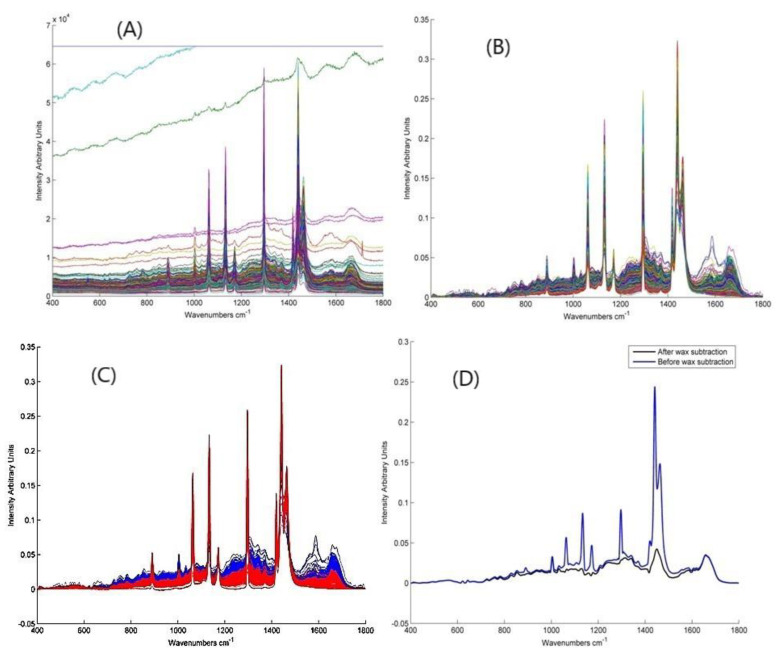
Spectral processing steps (**A**) Raw spectra. (**B**) Spectra after first quality control step, smoothing, baseline correction and normalization. (**C**) Spectra after k-means grouping; the spectra in red have high wax and low biological content while those in blue have higher biological content and less wax. (**D**) Spectra after glass and wax subtraction.

All the data analysis was carried out using MATLAB (Mathworks, Natick, MA, US) with the PLS-Toolbox (Eigenvector Research Inc., Manson, WA, USA) and in-house algorithms. Two quality control steps were employed (Figure 7). In the first, before processing, spectra with excess scatter/background were eliminated by setting a maximum intensity. Subsequent processing involved smoothing with a Savitsky Golay filter (5th order, 13 points) then correcting the baseline with a rubberband function, and finally vector normalisation. The second quality control step involved removing the spectra with excess wax and low biological content. This was achieved using k-means clustering which is used to partition data into groups such that variation is minimised within groups but maximised between groups. It assigns data points to their closest centre points which are changed with each iteration until optimal convergence is met. The next step was digitally subtracting the wax and glass backgrounds; which was carried out using the non-negatively constrained least squares fitting (NNLS) method. A PCA of the epithelium and connective tissue has demonstrated that the primary (~60%) variance of spectra of both FFPP tissue types derives from the contribution of the paraffin wax [43]. Therefore, for a detailed analysis of the more subtle biochemical origins of potentially malignancy, the contributions of the paraffin to the spectra were removed. A matrix of 300 wax and glass spectra were used as inputs for the NNLS along with spectra of pure cell components such as DNA and RNA. Using a matrix, instead of a mean spectrum, accounts for the inhomogeneity in the wax spectra which is a result of the microcrystalline domains being randomly oriented with respect to the laser source [43].

Partial least squares discriminant analysis (PLSDA) was used to build the classifier. It is a supervised form of multivariate analysis that works as a linear classifier that aims to separate the data into groups using a hyperplane. It is a generalisation of multiple linear regression (MLR), in which a set of dependent variables y is regressed against independent predictor variables X. Similar to linear discriminate analysis (LDA), it aims to maximise the variance between groups and minimise the variance within groups. It is based on partial least Squares Regression (PLSR). Whereas, in classic PLSR, y is a matrix of continuous variables, in PLSDA it is categorical and used to assign the observations into classes. The data was divided into y classes from 1 to 5, corresponding to benign, mild, moderate and severe dysplasia and SCC. Similar y class assignments were made according to gender, smoking status etc. The loadings of the discriminate hyperplanes or latent variables (LV)s were plotted to give more information on the source of the variance. While it is similar to other statistical methods such as PCA, the PLSDA LVs are calculated to maximise the covariance between the spectral variation and group/category so that the LVs explain the diagnostically relevant variations rather than the most prominent variations in the spectral dataset. Leave one patient out cross validation (LOPOCV) was used as a cross validation method to avoid overtraining the model. In LOPOCV, the spectra of all but one patient are used as a training set and a prediction is made for the left out patient. This is repeated so that the spectra of each patient are left out and predicted once.

Receiver operating characteristic (ROC) curves were plotted for each class. ROCs are a plot of the true positive rate (sensitivity) against the false positive rate (1-specificity) over a continuous range (from 0 to 1) of cut off points of a classifier. Each point on the ROC curve represents a sensitivity/specificity pair corresponding to a particular decision threshold. Accuracy is measured by the area under the ROC curve (AUC), so that, the closer the curve tends to the left and top borders, the more accurate the classifier. Conversely, the closer the curve is to the diagonal (baseline), the higher the misclassification rate and the lower the accuracy. The baseline is at 0.5, while a perfect classifier would have an AUC of 1. In general, an AUC of 0.5 is considered to have no discrimination, while 0.7 to 0.8 is considered acceptable, 0.8 to 0.9 is considered excellent, while over 0.9 is considered outstanding [44]. Different approaches to estimate the ROC curve lead to different estimates of the AUC. Both the estimated AUC (using the whole dataset) and cross validated AUC (leaving one patient dataset out in each iteration) are shown.

## 5. Conclusions

The finding that Raman spectroscopy can differentiate between cancer and dysplasia is very important, as the management and prognosis is different for both. Dysplasia is a common finding in tumour borders and regenerative changes which mimic dysplasia can often be found in the margins of resected tumours [45]. The balance between being conservative and maintaining as much of the tissue as possible, which is important both aesthetically and functionally, and removing enough of the tumour to prohibit recurrence is a difficult one in oral cancer surgery. Hence the ability of Raman spectroscopy to discriminate between cancerous and dysplastic and/or healthy tissue can be important in striking that balance. The finding that smoking and the presence of inflammation have a significant impact on the Raman classification highlights the importance of accounting for these variables in any future studies to be able to develop more robust diagnostic algorithms.

## Figures and Tables

**Table 1:** Tentative peak assignments, adapted from Movasaghi et al. [30].

Wavenumber (cm^−1^)	Assignment
484–490	Glycogen
599/600	Nucleotide conformation
666	G, T (ring breathing modes in DNA bases)
752	Symmetric breathing mode of tryptophan
782	DNA
811/812	RNA O-P-O stretch
814	C-C stretching (collagen assignment)
838	Deformative vibrations of amine groups
855	Ring breathing in tyrosine/C-C stretching in proline
919	C-C stretch of Proline ring/glucose lactic acid C-C, proline ring (collagen assignment)
934/935	Protein/C-C backbone (collagen assignment)
937/8	Proline, hydroxyproline (C-C) skeletal of collagen backbone
1001/1002	Phenylalanine ring breathing
1030–34	Phenylalanine of collagen
1128/1129	Skeletal C-C stretch in lipids
1131	Fatty acid
1237	Amide III
1245–1248	Amide III of collagen
1265	Amide III
1278	Proteins including collagen I
1285	Differences in collagen
1315–1317	Guanine
1333	Guanine
1336	Polynucleotide chain (DNA purine bases)
1368	Guanine TRP protein, porphrin, lipids
1373	T, A, G (ring breathing modes of the DNA/RNA bases)
1437	CH_2_ deformation (lipid)
1441	Wax
1449/1450	C-H vibration lipids
1460	CH_2_/CH_3_ deformation in Lipids
1554	Amide II
1572–1578	Guanine adenine
1650	Amide I
1652–1655	Lipid C=C (lipids)/Amide I
1666–1668	Protein/collagen
1674	C=C stretch in cholesterol
1700–1750	Amino acids aspartic and glutamic acid

**Table 2:** Sensitivity and specificity values obtained from PLSDA classification with LOPOCV *.

Pathology	Epithelium		Connective Tissue	
	Sensitivity (%)	Specificity (%)	Sensitivity (%)	Specificity (%)
Benign	74	49	81	44
Mild	67	38	67	46
Moderate	39	86	42	61
Severe	69	57	59	67
SCC	65	76	88	72

LOPOCV * = Leave one patient out cross validation.

**Table 3:** Sensitivity and specificity values from PLSDA with LOPOCV for smoking status in epithelium.

Statistic	Non-Smoker (*n* = 13)	Ex-Smoker (*n* = 17)	Smoker (*n* = 13)
Sensitivity (%)	83	81	52
Specificity (%)	46	38	88

**Table 4:** Number of inflamed samples per class.

Class	Benign (*n* = 17)	Mild (*n* = 20)	Moderate (*n* = 20)	Severe (*n* = 10)	SCC (*n* = 5)
Number Inflamed	2	3	9	7	5

## Data Availability

The data that support the findings of this study are available from the corresponding author upon reasonable request.

## Supplementary Materials

The following are available online at https://www.mdpi.com/2072-6694/13/4/619/s1, Figure S1: ROC curves for (A) Benign (B) Mild (C) Moderate (D) Severe and (E) SCC epithelial tisssues. Figure S2: ROC curves for (A) Benign (B) Mild (C) Moderate (D) Severe and (E) SCC connective tisssues. Figure S3: ROC curves for (A) Non-smoker (B) Ex-smokers and (C) Smokers epithelium. Figure S4: ROC curves for (A) Epithelium and (B) Connective tissue of Inflamed vs. Non-inflamed in all classes. Figure S5: ROC curves for (A) epithelium and (B) connective tissue of inflamed vs. non-inflamed in the moderately dysplastic lesions. Figure S6: ROC curves for (A) Epithelium and (B) Connective tissue of Female vs. Male.

## References

[1] Bray F., Ferlay J., Soerjomataram I., Siegel R.L., Torre L.A., Jemal A. (2018). Global cancer statistics 2018: GLOBOCAN estimates of incidence and mortality worldwide for 36 cancers in 185 countries. CA A Cancer J. Clin..

[2] Pfeifer G.P., Denissenko M.F., Olivier M., Tretyakova N., Hecht S.S., Hainaut P. (2002). Tobacco smoke carcinogens, DNA damage and p53 mutations in smoking-associated cancers. Oncogene.

[3] Boffetta P., Hashibe M. (2006). Alcohol and cancer. Lancet Oncol..

[4] Hashibe M., Brennan P., Chuang S.C., Boccia S., Castellsague X., Chen C., Curado M.P., Dal Maso L., Daudt A.W., Fabianova E. (2009). Interaction between Tobacco and Alcohol Use and the Risk of Head and Neck Cancer: Pooled Analysis in the International Head and Neck Cancer Epidemiology Consortium. Cancer Epidemiol. Biomark. Prev..

[5] van der Waal I. (2009). Potentially malignant disorders of the oral and oropharyngeal mucosa; terminology, classification and present concepts of management. Oral Oncol..

[6] Silverman S. (2001). Demographics and occurrence of oral and pharyngeal cancers—The outcomes, the trends, the challenge. J. Am. Dent. Assoc..

[7] Marur S., Forastiere A.A. (2008). Head and neck cancer: Changing epidemiology, diagnosis, and treatment. Mayo Clin. Proc..

[8] Poh C.F., Ng S., Berean K.W., Williams P.M., Rosin M.P., Zhang L.W. (2008). Biopsy and histopathologic diagnosis of oral premalignant and malignant lesions. J. Can. Dent. Assoc..

[9] Abbey L.M., Kaugars G.E., Gunsolley J.C., Burns J.C., Page D.G., Svirsky J.A., Eisenberg E., Krutchkoff D.J., Cushing M. (1995). Intraexaminer and interexaminer reliability in the diagnosis of oral epithelial dysplasia. Oral Surg. Oral Med. Oral Pathol. Oral Radiol. Endod..

[10] Scully C. (2014). Challenges in predicting which oral mucosal potentially malignant disease will progress to neoplasia. Oral Dis..

[11] Puppels G.J., Demul F.F.M., Otto C., Greve J., Robertnicoud M., Arndtjovin D.J., Jovin T.M. (1990). Studying single living cells and chromosomes by confocal raman microspectroscopy. Nature.

[12] Jermyn M., Desroches J., Aubertin K., St-Arnaud K., Madore W.J., De Montigny E., Guiot M.C., Trudel D., Wilson B.C., Petrecca K. (2016). A review of Raman spectroscopy advances with an emphasis on clinical translation challenges in oncology. Phys. Med. Biol..

[13] Santos I.P., Barroso E.M., Schut T.C.B., Caspers P.J., van Lanschot C.G.F., Choi D.H., van der Kamp M.F., Smits R.W.H., van Doorn R., Verdijk R.M. (2017). Raman spectroscopy for cancer detection and cancer surgery guidance: Translation to the clinics. Analyst.

[14] Upchurch E., Isabelle M., Lloyd G.R., Kendall C., Barr H. (2018). An update on the use of Raman spectroscopy in molecular cancer diagnostics: Current challenges and further prospects. Expert Rev. Mol. Diagn..

[15] Hiremath G., Locke A., Sivakumar A., Thomas G., Mahadevan-Jansen A. (2019). Clinical translational application of Raman spectroscopy to advance Benchside biochemical characterization to bedside diagnosis of esophageal diseases. J. Gastroenterol. Hepatol..

[16] Hubbard T.J.E., Shore A., Stone N. (2019). Raman spectroscopy for rapid intra-operative margin analysis of surgically excised tumour specimens. Analyst.

[17] Kumar P., Ingle A., Krishna C.M. In vivo Raman spectroscopy: Monitoring cancer progression post carcinogen withdrawal. Proceedings of the Conference on Optical Imaging, Therapeutics, and Advanced Technology in Head and Neck Surgery and Otolaryngology.

[18] Santana-Codina N., Marce-Grau A., Muixi L., Nieva C., Marro M., Sebastian D., Munoz J.P., Zorzano A., Sierra A. (2019). GRP94 Is Involved in the Lipid Phenotype of Brain Metastatic Cells. Int. J. Mol. Sci..

[19] Chrabaszcz K., Kochan K., Fedorowicz A., Jasztal A., Buczek E., Leslie L.S., Bhargava R., Malek K., Chlopicki S., Marzec K.M. (2018). FT-IR- and Raman-based biochemical profiling of the early stage of pulmonary metastasis of breast cancer in mice. Analyst.

[20] Farhane Z., Nawaz H., Bonnier F., Byrne H.J. (2018). In vitro label-free screening of chemotherapeutic drugs using Raman microspectroscopy: Towards a new paradigm of spectralomics. J. Biophotonics.

[21] Sahu A., Krishna C.M. (2017). Optical diagnostics in oral cancer: An update on Raman spectroscopic applications. J. Cancer Res. Ther..

[22] Carvalho L., Bonnier F., O'Callaghan K., O’Sullivan J., Flint S., Byrne H.J., Lyng F.M. (2015). Raman micro-spectroscopy for rapid screening of oral squamous cell carcinoma. Exp. Mol. Pathol..

[23] Carvalho L., Bonnier F., Tellez C., dos Santos L., O’Callaghan K., O’Sullivan J., Soares L.E.S., Flint S., Martin A.A., Lyng F.M. (2017). Raman spectroscopic analysis of oral cells in the high wavenumber region. Exp. Mol. Pathol..

[24] Yu M.X., Yan H., Xia J.B., Zhu L.Q., Zhang T., Zhu Z.H., Lou X.P., Sun G.K., Dong M.L. (2019). Deep convolutional neural networks for tongue squamous cell carcinoma classification using Raman spectroscopy. Photodiagnosis Photodyn. Ther..

[25] Jeng M.J., Sharma M., Sharma L., Chao T.Y., Huang S.F., Chang L.B., Wu S.L., Chow L. (2019). Raman Spectroscopy Analysis for Optical Diagnosis of Oral Cancer Detection. J. Clin. Med..

[26] Cals F.L.J., Schut T.C.B., Caspers P.J., de Jong R.J.B., Koljenovic S., Puppels G.J. (2018). Raman spectroscopic analysis of the molecular composition of oral cavity squamous cell carcinoma and healthy tongue tissue. Analyst.

[27] Cals F.L.J., Koljenovic S., Hardillo J.A., de Jong R.J.B., Schut T.C.B., Puppels G.J. (2016). Development and validation of Raman spectroscopic classification models to discriminate tongue squamous cell carcinoma from non-tumorous tissue. Oral Oncol..

[28] Barroso E.M., ten Hove I., Schut T.C.B., Mast H., van Lanschot C.G.F., Smits R.W.H., Caspers P.J., Verdijk R., Hegt V.N., de Jong R.J.B. (2018). Raman spectroscopy for assessment of bone resection margins in mandibulectomy for oral cavity squamous cell carcinoma. Eur. J. Cancer.

[29] Cals F.L.J., Schut T.C.B., Hardillo J.A., de Jong R.J.B., Koljenovic S., Puppels G.J. (2015). Investigation of the potential of Raman spectroscopy for oral cancer detection in surgical margins. Lab. Investig..

[30] Movasaghi Z., Rehman S., Rehman I.U. (2007). Raman spectroscopy of biological tissues. Appl. Spectrosc. Rev..

[31] Kalluri R., Weinberg R.A. (2009). The basics of epithelial-mesenchymal transition. J. Clin. Investig..

[32] Speight P.M. (2007). Update on Oral Epithelial Dysplasia and Progression to Cancer. Head Neck Pathol..

[33] Sahu A., Deshmukh A., Ghanate A.D., Singh S.P., Chaturvedi P., Krishna C.M. (2012). Raman Spectroscopy of Oral Buccal Mucosa: A Study on Age-Related Physiological Changes and Tobacco-Related Pathological Changes. Technol. Cancer Res. Treat..

[34] Depciuch J., Sowa-Kucma M., Nowak G., Dudek D., Siwek M., Styczen K., Parlinska-Wojtan M. (2016). Phospholipid-protein balance in affective disorders: Analysis of human blood serum using Raman and FTIR spectroscopy. A pilot study. J. Pharm. Biomed. Anal..

[35] Singh S.P., Deshmukh A., Chaturvedi P., Krishna C.M. (2012). In vivo Raman spectroscopic identification of premalignant lesions in oral buccal mucosa. J. Biomed. Opt..

[36] Rashid N., Nawaz H., Poon K.W.C., Bonnier F., Bakhiet S., Martin C., O’Leary J.J., Byrne H.J., Lyng F.M. (2014). Raman microspectroscopy for the early detection of pre-malignant changes in cervical tissue. Exp. Mol. Pathol..

[37] Sorsa T., Tjaderhane L., Salo T. (2004). Matrix metalloproteinases (MMPs) in oral diseases. Oral Dis..

[38] Mashhadiabbas F., Fayazi-Boroujeni M. (2017). Correlation of vascularization and inflammation with severity of oral leukoplakia. Iran. J. Pathol..

[39] Negus R.P.M., Stamp G.W.H., Hadley J., Balkwill F.R. (1997). Quantitative assessment of the leukocyte infiltrate in ovarian cancer and its relationship to the expression of C-C chemokines. Am. J. Pathol..

[40] Talmadge J.E. (2011). Immune cell infiltration of primary and metastatic lesions: Mechanisms and clinical impact. Semin. Cancer Biol..

[41] Takahashi H., Ogata H., Nishigaki R., Broide D.H., Karin M. (2010). Tobacco Smoke Promotes Lung Tumorigenesis by Triggering IKK beta- and JNK1-Dependent Inflammation. Cancer Cell.

[42] Feller L., Altini M., Lemmer J. (2013). Inflammation in the context of oral cancer. Oral Oncol..

[43] Ibrahim O., Maguire A., Meade A.D., Flint S., Toner M., Byrne H.J., Lyng F.M. (2017). Improved protocols for pre-processing Raman spectra of formalin fixed paraffin preserved tissue sections. Anal. Methods.

[44] Mandrekar J.N. (2010). Receiver Operating Characteristic Curve in Diagnostic Test Assessment. J. Thorac. Oncol..

[45] Cankovic M., Ilic M.P., Vuckovic N., Bokor-Bratic M. (2013). The histological characteristics of clinically normal mucosa adjacent to oral cancer. J. Cancer Res. Ther..

