# Supplementary material for: The Potential of Raman Spectroscopy in the Diagnosis of Dysplastic and Malignant Oral Lesions
Source: Cancers (Basel). 2021 Feb 4;13(4):619. doi: 10.3390/cancers13040619 (PMC7913942; doi:10.3390/cancers13040619)
Supplement: Supplementary file 1 [file cancers-13-00619-s001.pdf]

cancers

MDPI

Supplemental Materials

The Potential of Raman Spectroscopy in the Diagnosis of Dysplastic and Malignant Oral Lesions

Ola Ibrahim, Mary Toner, Stephen Flint, Hugh J. Byrne and Fiona M. Lyng

Supplementary figures for; The potential of Raman spectroscopy in the diagnosis of dysplastic and malignant oral lesions

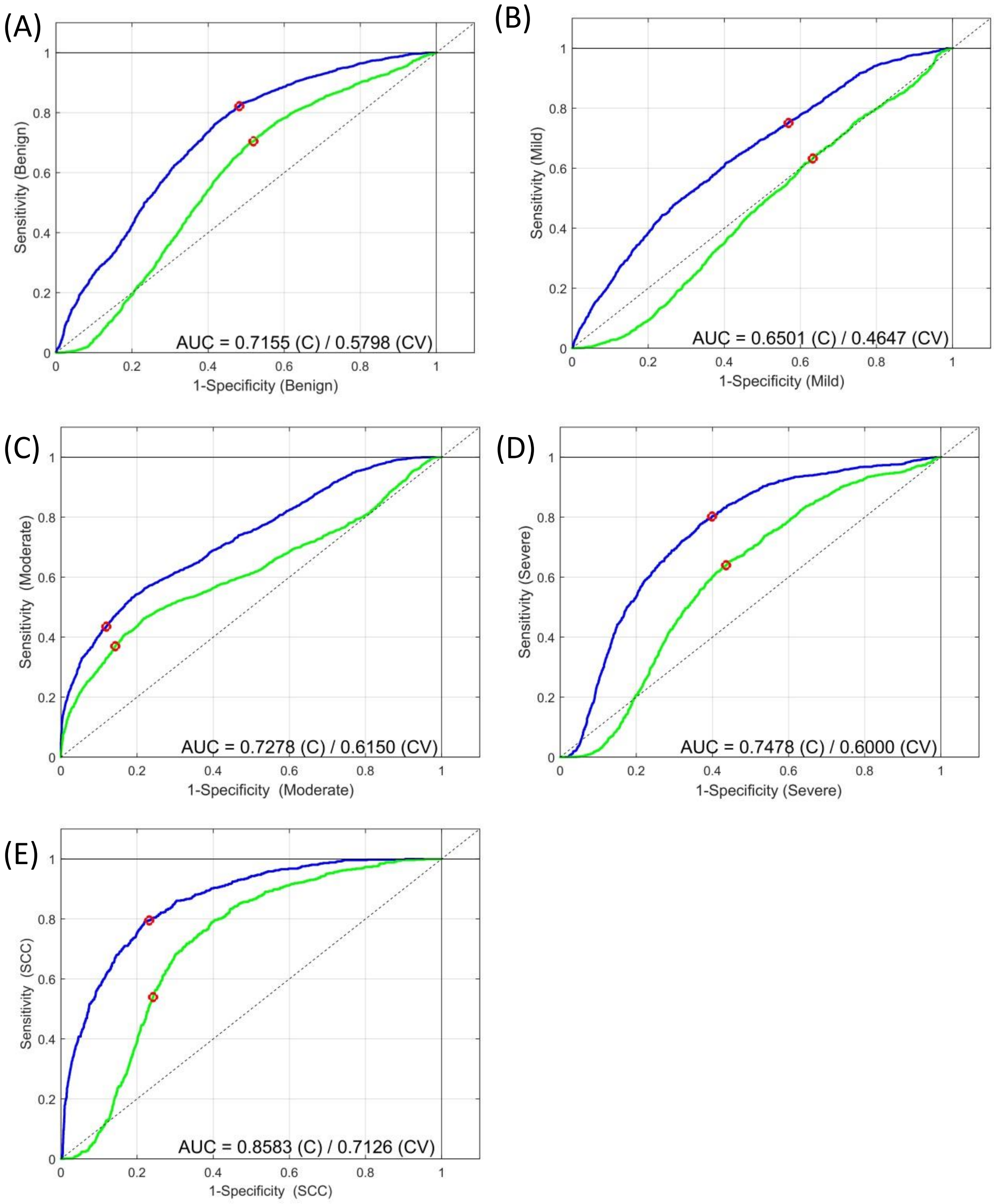

Figure S1. ROC curves for (A) Benign (B) Mild (C) Moderate (D) Severe and (E) SCC epithelial tisssues. The blue line is the estimated and the green line is the cross validated ROC curve. Area under the curve (AUC) is a measure of the accuracy of the classifier, C is the calibrated and CV is the cross validated AUC. The red dot(s) represents the calculated sensitivity and 1-specificty on the y and x axis respectively.

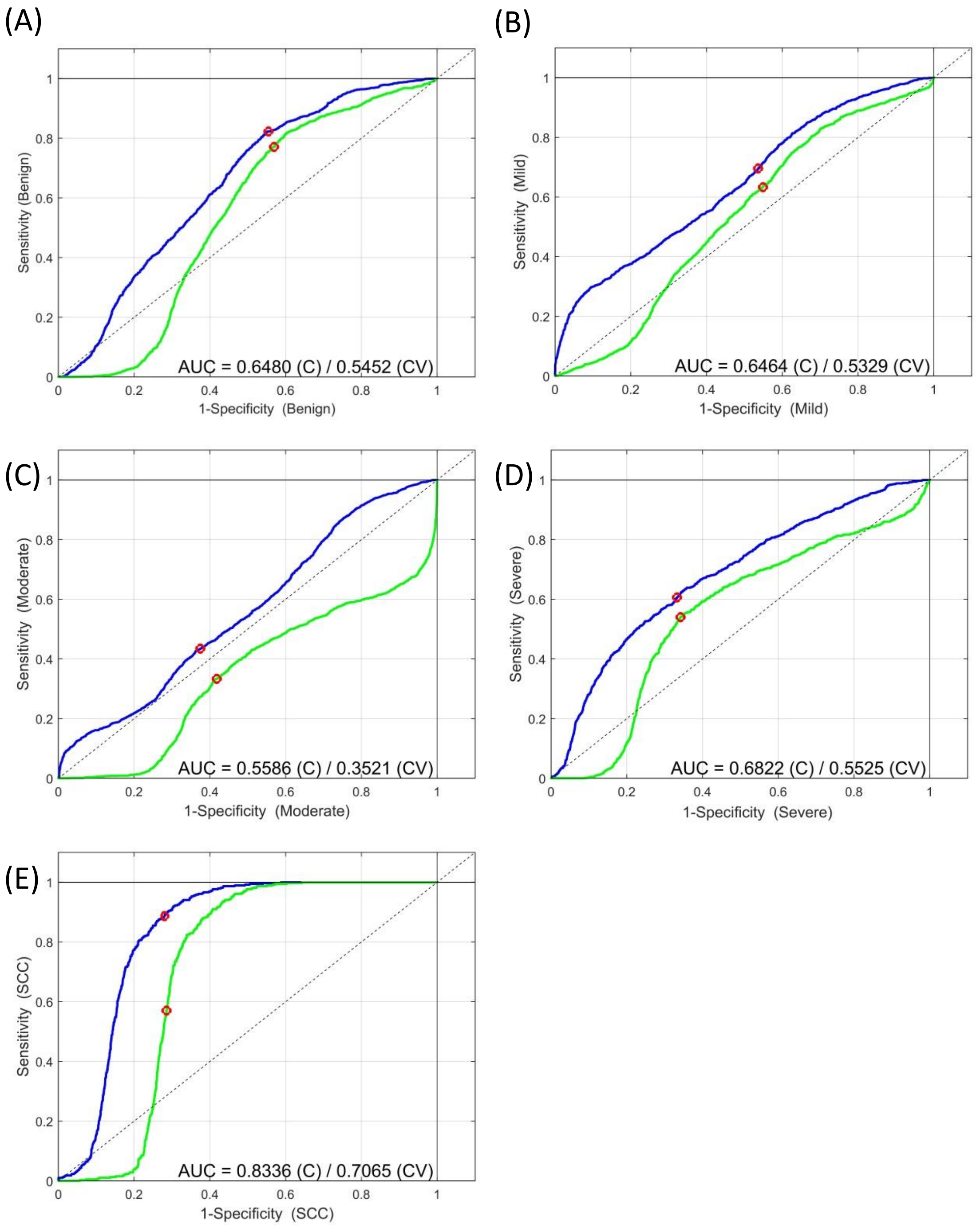

Figure S2 ROC curves for (A) Benign (B) Mild (C) Moderate (D) Severe and (E) SCC connective tisssues. The blue line is the estimated and the green is the cross validated ROC curve. AUC is a measure of the accuracy of the classifier.

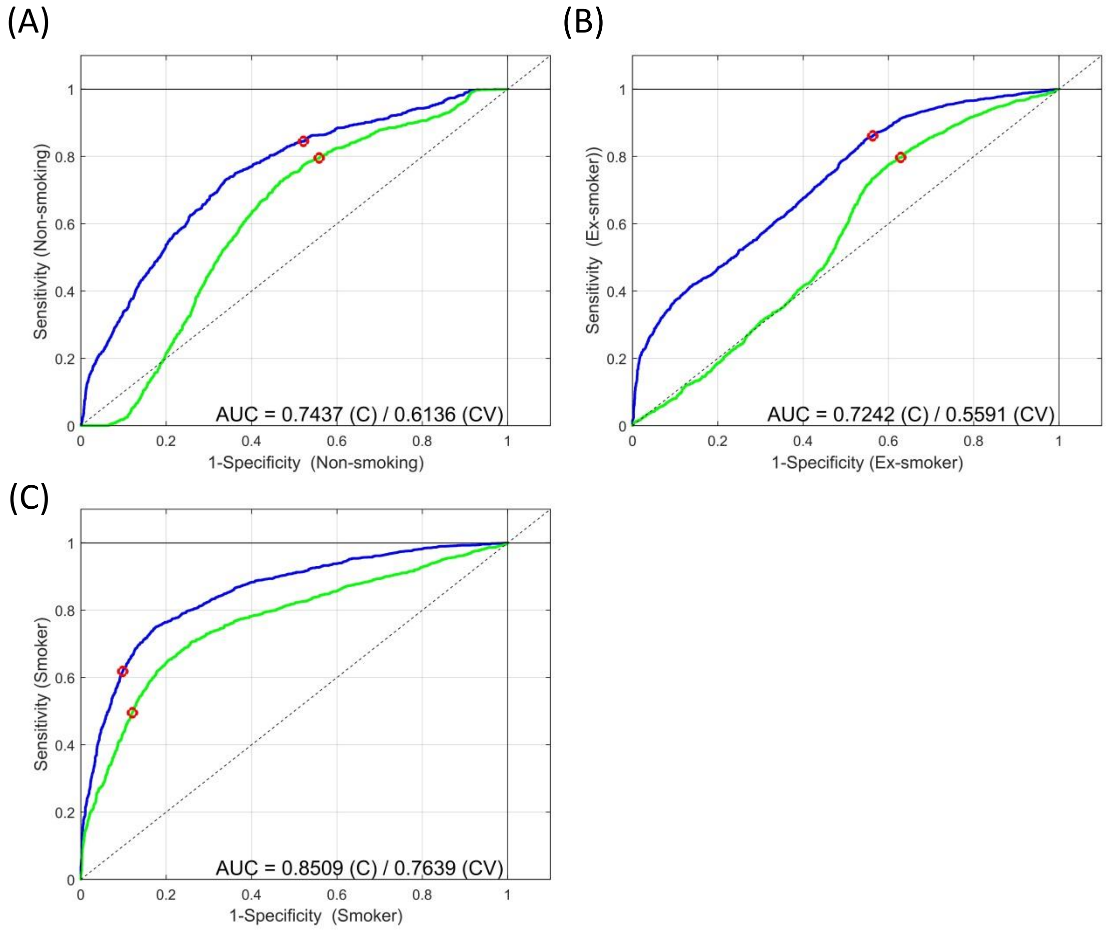

Figure S3 ROC curves for (A) Non-smoker (B) Ex-smokers and (C) Smokers epithelium. The blue line is the estimated and the green line is the cross validated ROC curve. AUC is a measure of the accuracy of the classifier, C is the calibrated and CV is the crossvalidated AUC.

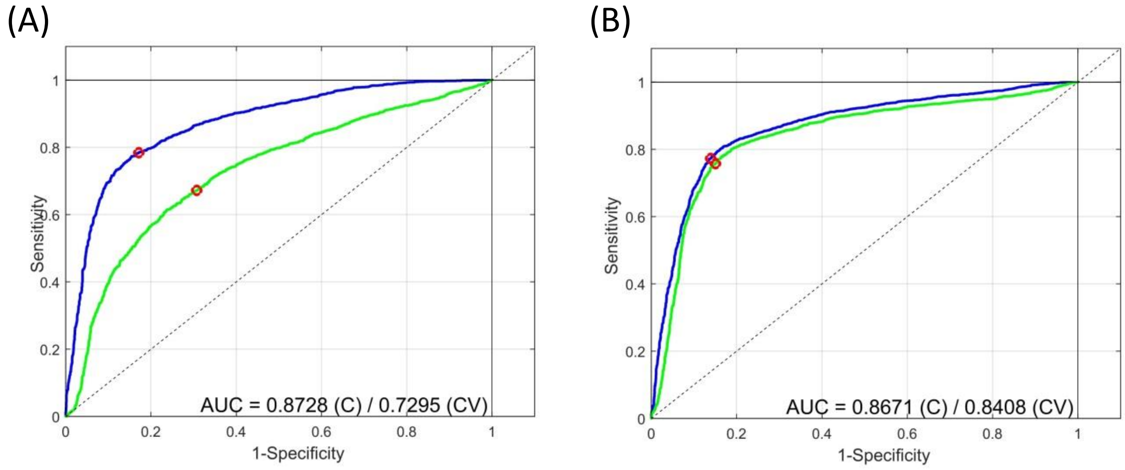

Figure S4 ROC curves for (A) Epithelium and (B) Connective tissue of Inflamed vs Non-inflamed in all classes. The blue line is the estimated and the green the cross validated ROC curve. AUC is a measure of the accuracy of the classifier, (C) is the calibrated and (CV) is the crossvalidated AUC.

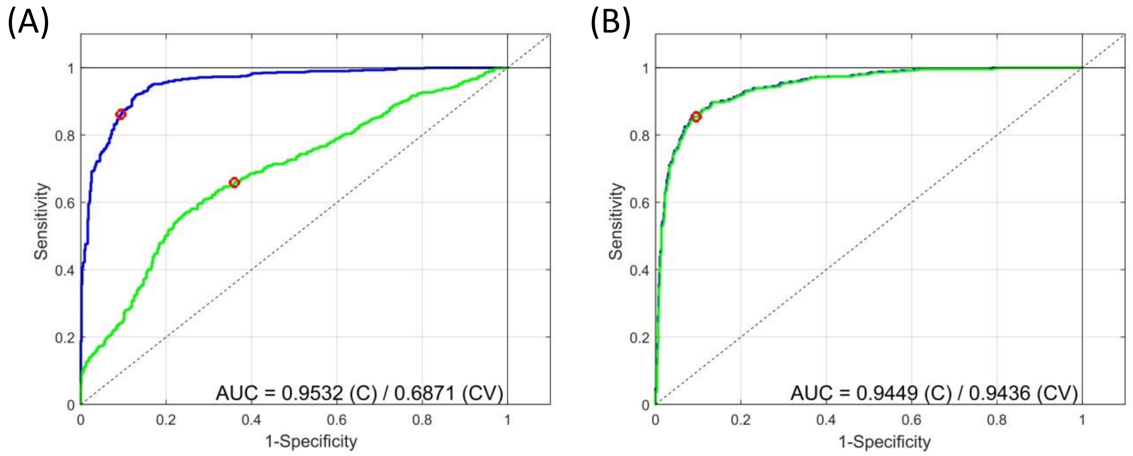

Figure S5 ROC curves for (A) epithelium and (B) connective tissue of inflamed vs non-inflamed in the moderately dysplastic lesions. The blue line is the estimated and the green line is the cross validated ROC curve. AUC is a measure of the accuracy of the classifier, C is the calibrated and CV is the crossvalidated AUC.

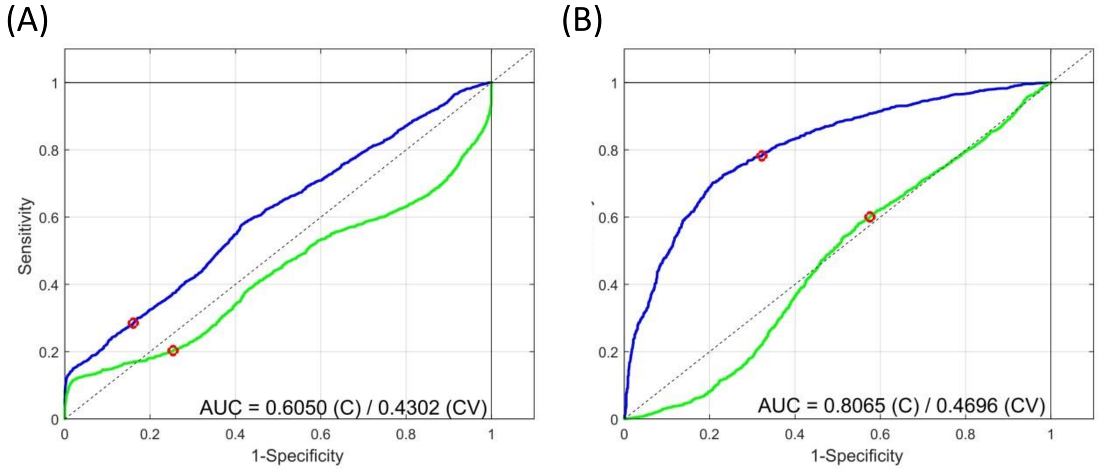

Figure S6 ROC curves for (A) Epithelium and (B) Connective tissue of Female vs Male. The blue line is the estimated and the green the cross validated ROC curve. AUC is a measure of the accuracy of the classifier, (C) is the calibrated and (CV) is the crossvalidated AUC.
